# Survival of Patients With Idiopathic Inflammatory Myopathies in Slovenia

**DOI:** 10.3389/fmed.2021.801078

**Published:** 2021-12-20

**Authors:** Alojzija Hočevar, Andrej Viršček, Monika Krošel, Suzana Gradišnik, Žiga Rotar, Matija Tomšič, Iztok Holc

**Affiliations:** ^1^Department of Rheumatology, University Medical Centre Ljubljana, Ljubljana, Slovenia; ^2^Faculty of Medicine, University of Ljubljana, Ljubljana, Slovenia; ^3^Statistical Office of the Republic of Slovenia, Ljubljana, Slovenia; ^4^Department of Rheumatology, University Medical Centre Maribor, Maribor, Slovenia; ^5^Faculty of Medicine, University of Maribor, Maribor, Slovenia

**Keywords:** inflammatory myopathy, prognostic factors, survival, mortality, cancer

## Abstract

**Background:** Idiopathic inflammatory myopathies (IIMs) are rare systemic diseases associated with significant morbidity and mortality. The aim of our study was to estimate for the first time the survival of IIM patients in Slovenia.

**Methods:** We included IIM patients diagnosed between January 2005 and December 2020 and followed at two secondary/tertiary rheumatology centers in the country. To study survival/mortality the censor date of April 14 2021 was set. Kaplan–Meier analysis and standardized mortality ratio (SMR) were plotted using data of age and sex matched Slovenian population as a reference. Cox proportional hazards regression analysis was used to study prognostic factors for IIM mortality.

**Results:** During the 16-year observation period, we identified 217 new IIM patients. During follow up 65 (30.0%) patients died. In the first year following IIM diagnosis the SMR was nearly 7-fold higher compared to the matched general population [SMR 6.88 (95%CI 4.41–10.24)] and remained higher also during the following 4 years. However, when excluding IIM patients with cancer, the survival outcome was, except in the first year after IIM diagnosis [SMR 5.55 (95%CI 3.10–9.15)], comparable to matched general population. In addition to cancer [HR 3.71 (95% CI 2.18–6.04)], cardiac involvement [HR 2.18 (95% CI 1.07–4.45)], fever [HR 2.13 (95% CI 1.13–4.03)], and older age [HR 1.07 (95% CI 1.04–1.09)] were extracted as prognostic factors associated with death.

**Conclusion:** The survival of patients with IIM patients was substantially worse compared to matched general population. Cancer was the leading cause of death in our cohort.

## Introduction

The group of idiopathic inflammatory myopathies (IIM) represents rare and heterogeneous systemic autoimmune diseases, characterized by a progressive and predominantly proximal muscle weakness. In addition, IIM patients may manifest with constitutional symptoms, skin, articular, lung, heart, gastrointestinal involvement, or vasculopathy. The multi-organ nature of IIMs is associated with significant morbidity and disability (1).

Data on survival of IIM are limited and show wide variations. These differences likely reflect variations in studies' timeframe, design, and heterogeneity of the included population e.g., different classification criteria used, population vs. hospital-based studies, variations in the follow up time. In historic cohorts 1- and 5-year survival of 72% and 52–65% were reported (2, 3). Higher survival was found in studies that excluded cancer-associated myositis or had a greater proportion of juvenile IIM cases compared to studies that included patients with higher age at diagnosis, significant diagnostic or treatment delay, significant respiratory muscle involvement, interstitial lung disease and/or cancer (4, 5). Furthermore, in the recent years with a better IIMs management, an improved survival compared to historical cohorts was found (6–8). In a Spanish cohort, Nuño-Nuño et al. reported a 5- and 10-year survival rate of 87 and 77%, respectively (6). Similarly, Johnson et al. found a considerably improved 1-, 5-, and 10-year survival of IIM patients compared to earlier reports, even in patients with interstitial lung disease, the latter being recognized as a prognostically unfavorable factor of increased mortality in IIMs (7).

Immune mediated necrotizing myopathy represents a relatively recently recognized IIM entity which prognosis (9) has not been specifically evaluated in above cited survival analysis (2–7). However Allenbach et al. showed in their study a poor prognosis in necrotizing myopathy associated with cancer compared to non-cancer associated necrotizing myopathy (10).

Data on prognosis and survival of IIM patients for our country is lacking. In the presenting retrospective longitudinal two-center study, we performed for the first time the nationwide analysis of survival/mortality in adult IIM cohort, thereby considering currently recognized IIM subtypes, including immune mediated necrotizing myopathy.

## Methods

### Setting

This retrospective study was conducted at the Department of Rheumatology, University Medical Center Ljubljana, and Department of Rheumatology, University Medical Center Maribor. Centers provide rheumatology services at secondary level to a population of around 720,000 and 273,00 adults, respectively, and represent the only tertiary centers in the country.

Adult patients (aged ≥ 18 years) with suspected inflammatory myopathy are generally managed by rheumatologists, and commonly referred to these two centers by general practitioners or by other subspecialists (e.g., neurologists, pulmonologists, dermatologists, and oncologists).

Patients were ascertained by searching the electronic medical records for International Statistical Classification of Diseases 10th revision codes: M33, M35.1, M35.8, M60, G72, G73, J84, and by checking the list of patients with performed muscle biopsies, provided from the Institute of Pathology, Medical Faculty Ljubljana.

### Patient Selection and Workup in IIM

We included adult patients with IIM diagnosed for the first time in the period between January 2005 and December 2020 and followed at one of both centers.

The diagnosis of IIM was based on clinical history and examination, laboratory investigations, functional tests, imaging and/or histopathological findings. Bohan and Peter classification criteria and/or 2017 European League Against Rheumatism/American College of Rheumatology classification criteria for adult and juvenile idiopathic inflammatory myopathies, as well as European Neuromuscular Center 2004 guidelines helped with clinical decision making (9, 11–13). Following the diagnosis, we further subclassified IIM patients into six subgroups: polymyositis, dermatomyositis, antisynthetase syndrome, myositis overlaps with other connective tissue disease, immune mediated necrotizing myopathy and inclusion body myositis.

A detailed baseline evaluation protocol for myositis was routinely followed and consisted of a structured history, an extensive laboratory workup (including a panel of myositis specific antibodies against Jo-1, PL-7, PL-12, EJ, Mi-2, MDA5, TIF1, NXP2, SAE, HMGCR antigens, and a panel of myositis-associated antibodies including Ro52, Ku, and Pm/Scl, among others), functional tests (a standardized assessment of muscle weakness, electromyography). In patients with laboratory and/or functional signs of myopathy, muscle biopsy was performed. Muscle biopsies were evaluated using bright field microscopy, direct immunofluorescence and assortment of biochemical stainings at pathologist's discretion.

A high-resolution computed tomography (HRCT) was performed when indicated based on the clinical presentation and the findings of routinely performed chest X-ray and pulmonary function tests. Cardiac involvement was assessed by laboratory investigations (troponin, NT-pro-BNP), electrocardiogram and echocardiography. All patients underwent cancer screening.

For the purpose of the study, two assessors (AH, IH) reviewed in detail medical records of all IIM patients included in the study.

During the follow up, disease activity was assessed clinically using laboratory investigations (e.g., muscle enzymes and inflammatory parameters), functional tests (e.g., a pulmonary function test and assessment of muscle weakness) and imaging (e.g., high resolution lung computer tomography).

### Mortality of IIM Patients During Follow-Up and Prognostic Factors

The primary endpoint was overall patient death. To study survival a censor date April 14, 2021, was selected. Patients were followed until death or the censor date, whichever came first. Survival/mortality was assessed using the Kaplan-Meier analysis (14).

We compared IIM mortality with the mortality of age and sex matched Slovenian population obtained from the Department of Demographic and Social Statistics at the Statistical Office of the Republic of Slovenia^1^.

Medical records of patients who died were analyzed to ascertain the causes of death. For patients who died outside the UMCs, the causes of death were determined *via* tracing the medical records of the general practitioner, other hospital and/or emergency unit where the patients died.

The prognostic role of selected variables (demographic data) (sex, age), symptom duration time (from symptom onset until the IIM diagnosis), IIM subgroups, specific IIM clinical characteristics (involvement of individual organs/organ systems, immunoserology), and intensity of induction immunomodulatory treatment in predicting mortality was determined.

### Statistical Analysis

The results were expressed as a median and interquartile range (IQR) for metric, and as proportions for categorical variables. To test the differences between alive and deceased IIM patients we used the Mann-Whitney test for metric, and Fisher's exact-test for categorical variables. Kaplan–Meier analysis and a standardized mortality ratio (SMR) were used to analyze survival/mortality. Prognostic factors were extracted using Cox proportional hazards regression analysis. The significance threshold selected in all analyses was set at 0.05.

### Ethics Committee Approval

The study was approved by the National medical ethics committee, approval number 99/04/15.

## Results

### IIM Patients

During the 16-year observation period, we identified 217 new IIM patients [152 (70.0%) females, median (interquartile) age 64.3 (52.4–72.8) years, range 22 to 94 years]. We diagnosed polymyositis, dermatomyositis, antisynthetase syndrome, other overlap IIMs, immune mediated necrotizing myopathy and inclusion body myositis in 25 (11.5%), 87 (40.1%), 50 (23.0%), 29 (13.4%), 24 (11.1%), and 2 (0.9%) patients, respectively. Muscle biopsy was performed in 189 (87.1%) patients and was consistent with the IIM in 171 (90.5%) of patients. Regarding the Bohan and Peter classification criteria, the criteria for definite and probable PM/DM were fulfilled in 48.8 and 72.8% of patients, whereas the ACR/ELUAR 2017 criteria for *definite IIM* with and without muscle biopsy were fulfilled in 54.0 and 48.4% of patients, respectively; and those for *probable IIM* with and without muscle biopsy in 71.4 and 70.5% of patients, respectively.

Fifteen (6.9%) patients had a history of cancer diagnosed within a year before diagnosed IIM and in 12 (5.5%) patients cancer was diagnosed concurrent with IIM. Clinical characteristics of our IIM cohort are presented in Table 1, column A.

**Table 1:** Clinical characteristics of patients with idiopathic inflammatory myopathy.

**Column A**		**Column B**		
**Characteristic**	**ALL IIM (217)**	**Deceased IIM (65)**	**Alive IIM (152)**	***P* value**
Sex (female)	152 (70.0%)	69.2%	70.4%	0.873
Age (years)^*^	64.3 (52.4–72.8)	71.1 (62.6–78.6)	59.7 (49.4–68.1)	<0.001
Symptom duration (m)^*^	4 (2–10)	3 (2–6)	4 (2–10)	0.088
Fever	30 (13.8%)	18.5%	11.8%	0.204
Arthritis	58 (26.7%)	18.5%	30.3%	0.094
Myositis	189 (87.1%)	92.3%	84.9%	0.184
Dysphagia	50 (23.0%)	30.8%	19.7%	0.082
Raynaud phenomenon	41 (18.9%)	9.2%	23.0%	0.022
Skin involvement	121 (55.8%)	58.5%	54.6%	0.656
Interstitial lung disease	71 (32.7%)	32.3%	32.9%	1.00
Number of cases with HRCT pattern NSIP/OP/NSIP+OP/UIP/undetermined	35/13/13/3/7	8/5/4/1/3	27/8/9/2/4	-
Cardiac involvement	25 (11.5%)	13.8%	10.5%	0.492
Cancer^**^	41 (18.9%)	41.5%	9.2%	<0.001
ANA positive	136 (62.7%)	66.2%	61.2%	0.542
anti-Jo1 positive	39 (18.0%)	21.5%	16.4%	0.127
Death	65 (30.0%)			
**IIM type**				
Polymyositis	25 (11.5%)	15.4%	9.9%	0.252
Dermatomyositis	87 (40.1%)	43.1%	38.3%	0.650
Antisynthetase syndrome	50 (23.0%)	24.6%	22.4%	0.003
Other overlap IIM	29 (13.4%)	4.6%	17.1%	0.015
Immune mediated necrotizing myopathy	24 (11.1%)	12.3%	10.5%	0.813
Inclusion body myositis	2 (0.9%)	0	1.3%	1.0

*IIM, Idiopathic inflammatory myopathy; ANA, antinuclear antibody*.

* *Median and interquartile range*.

** *Cancer diagnosed within a year before or concurrently with IIM or during follow up; HRCT, high resolution computer tomography; NSIP, non-specific interstitial pneumonia; OP, organizing pneumonia; UIP, usual interstitial pneumonia*.

### IIM Induction Treatment

We treated IIM patients in line with the local practice. Patients with predominant myositis were treated with systemic glucocorticoids with/without additional immunomodulatory drug(s). Methotrexate was most frequently used comedication, either as monotherapy, or in case of incomplete response as a combination with an additional immunomodulator. In case of interstitial lung involvement glucocorticoids were generally combined with cyclophosphamide. Intravenous immunoglobulins were prescribed as an add-on therapy in patients with dysphagia or severe refractory IIM course. Rituximab was also used as a rescue therapy in non-responders. In patients with active cancer baseline treatment was chosen in agreement with oncologists. Table 2 shows the frequency of different drug combinations used at baseline. Briefly, 205 (94.5%) patients received immunomodulatory treatment [64 (31.2%) glucocorticoid monotherapy, 133 (64.9%) glucocorticoids with additional immunomodulatory drug(s), and 8 (3.9%) patients immunomodulatory drug without a systemic glucocorticoid].

**Table 2:** Immunomodulatory treatment in patients with idiopathic inflammatory myopathy.

**Treatment**	**Patient no**.	**Treatment**	**Patient no**.
None	12 (5.5%)	GC + DMARD	133 (61.3%)
GC alone	64 (29.5%)	DMARD alone	8 (3.7%)
**DMARD**	**Patient no**.	**DMARD**	**Patient no**.
MTX	63	MTX+CNI	6
CyC	29	MTX+IVIG	4
AZA	6	CyC+CNI	2
MMF	3	CyC+IVIG	1
CNI	2	CyC+RTX	1
CQ	1	MTX+CNI+IVIG	3
IVIG	7	MTX+RTX+IVIG	2
RTX	6	MTX+AZA+IVIG	1
		MTX+CNI+RTX	1
		MTX+CNI+RTX+IVIG	3

*GC, glucocorticoids; DMARD, disease modifying antirheumatic drug; MTX, methotrexate; CyC, cyclophosphamide; AZA, azathioprine; MMF, mycophenolate mofetil; CNI, calcineurin inhibitor; CQ, chloroquine; IVIG, hyperimmune gammaglobulins; RTX, rituximab*.

### Survival/Mortality and Mortality Prognostic Factors

Patients were followed for a median of 59.6 (19.2–106.2) months, range 0.2–193 months. During the follow-up 65 (30.0%) patients died. One- and five-year survival rates were 88.9 and 75.5%, respectively. Fourteen (6.5%) patients developed *de novo* cancer during the follow-up. Furthermore, cancer was the most frequent cause of death in our cohort [22 (33.8%) patients], followed by cardiovascular diseases [19 (29.2%) patients] and infections [15 (23.1%) patients]. Active IIM was the least common cause of death [4 (6.4%) patients]. In 5 (7.7%) patients the cause of death was unknown. Table 1, column B presents the differences in clinical characteristics between surviving and deceased IIM patients.

**Figure 1:**
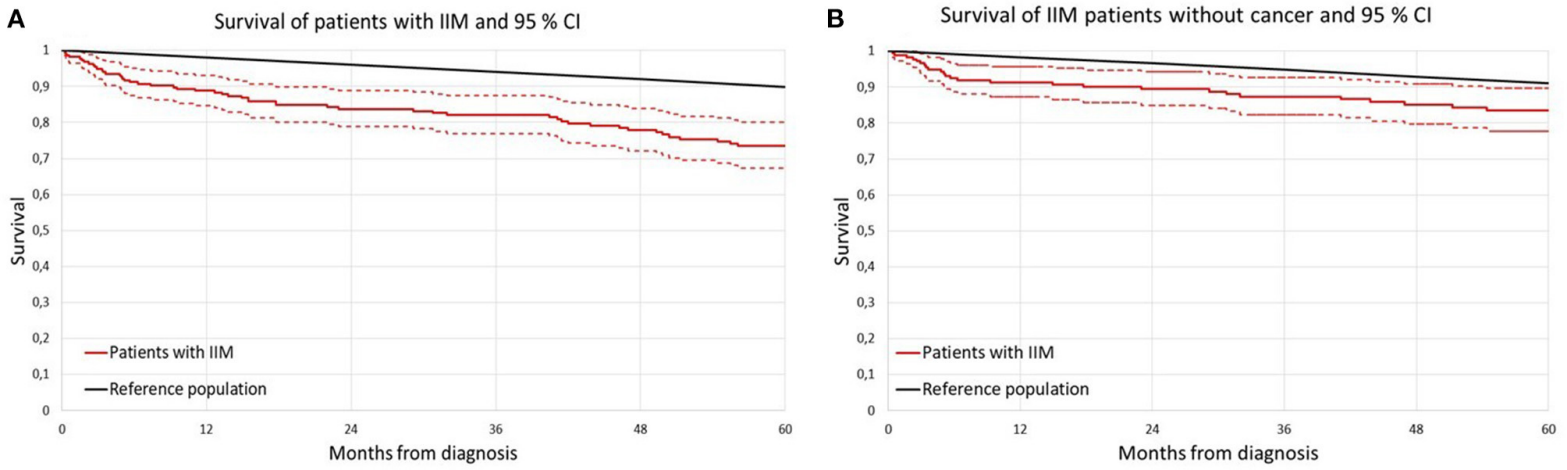
Survival curve of patients with idiopathic inflammatory myopathy (IIM) according to Kaplan-Meier analysis compared to sex and age matched general population. **(A)** Entire IIM cohort; **(B)** non-cancer IIM cohort; IIM idiopathic inflammatory myopathy.

Figure 1A shows the Kaplan-Meier survival curves of IIM patients and of age and sex matched general population as a comparator. IIM patients as a group had a significantly higher overall mortality compared to matched general population.

The results of Kaplan–Meier survival analysis after exclusion of IIM patients with cancer (cancer diagnosed either in a year preceding IIM, or concomitantly with IIM or during follow up) are presented in Figure 1B.

We found no significant sex related differences in the net survival estimates (15) during the first 5 years of follow-up either in entire IIM cohort (*p* = 0.297) nor in cancer excluded cohort (*p* = 0.768).

In Table 3, the SMRs in IIM patients are presented. Due to the limited number of patients followed beyond 5 years (only 108 patients) the results for the first 5 years of follow-up are shown only. In the 1st year following IIM diagnosis the SMR was 7-times higher compared to the general population [SMR 6.88 (95% CI 4.41–10.24)]. The risk remained higher during the following 4 years too. However, when excluding IIM patients with cancer from the analysis, the patients had an increased risk of death only in the 1st year following IIM diagnosis, but later on the survival was comparable to age and sex matched general population.

**Table 3:** The standardized mortality ratio of patients with idiopathic inflammatory myopathy.

**Follow up time**	**Observed deaths**	**Expected deaths**	**SMR (95% CI)**	***P* value**
**Entire IIM cohort**				
1 year	24	3.5	6.88 (4.41–10.24)	<0.001
2 years	10	3.1	3.23 (1.55–5.94)	0.003
3 years	3	2.8	1.06 (0.21–3.10)	0.929
4 years	7	2.8	2.46 (0.99–5.07)	0.053
5 years	7	2.3	3.00 (1.20–6.18)	0.021
**Cancer excluded IIM cohort**				
1 year	15	2.7	5.55 (3.10–9.15)	<0.001
2 years	3	2.4	1.23 (0.25–3.59)	0.881
3 years	3	2.3	1.33 (0.27–3.88)	0.783
4 years	3	2.3	1.32 (0.27–3.86)	0.790
5 years	2	2.0	0.98 (0.11–3.54)	0.665

*IIM, Idiopathic inflammatory myopathy; SMR, standardized mortality ratio; CI, confidence interval*.

In the Cox proportional hazards regression analysis cancer [HR 3.71 (95% CI 2.17–6.04); *p* < 0.001], cardiac involvement [HR 2.18 (95% CI 1.07–4.45); *p* = 0.034], fever [HR 2.13 (95% CI 1.13–4.03); *p* = 0.021] and age [HR 1.06 (95% CI 1.04–1.09); *p* < 0.001], were extracted as prognostic factors associated with death (Table 4). Disease duration prior to the diagnosis, patient sex, other clinical characteristics of IIM except fever and heart involvement, antinuclear antibody status or intensity of immunomodulatory treatment were not significantly associated with the risk of death. In a subgroup of IIM patients without cancer the Cox proportional hazards regression analysis revealed three risk factors associated with death: cardiac involvement [HR 2.76 (95% CI 1.18–6.42); *p* = 0.020], presence of anti-Jo1 antibody [HR 2.49 (95% CI 1.07–5.80); *p* = 0.035] and age [HR 1.09 (95% CI 1.06–1.12); *p* < 0.001].

**Table 4:** Predictors of death in patients with idiopathic inflammatory myopathy using Cox proportional hazards regression analysis.

**Covariate**	***P* value**	**Hazard ratio**	**95% confidence interval**
Cancer	<0.001	3.71	2.17–6.04
Cardiac involvement	0.034	2.18	1.07–4.45
Fever	0.021	2.13	1.13–4.03
Age	<0.001	1.06	1.04–1.09

## Discussion

IIMs are chronic systemic autoimmune disorders associated with increased mortality compared to general population. Nevertheless, major differences exist in reported mortality rates, depending on the study type, design, and characteristics of the analyzed IIM population. In the present two-center study, we estimated for the first time the overall mortality and mortality risk factors in a cohort of unselected consecutive clinically diagnosed adult IIM patients in Slovenia.

We found a 1- and 5-year survival rate of 88.9 and 75.5%, respectively. The overall mortality was nearly 7-fold higher in the 1st year following IIM diagnosis compared with the general population, and remained higher also during the following 4 years. Our results are in line with the study by Doblough et al. who noted almost 10-times higher mortality rate already within the 1st year of diagnosis compared with population (8).

Considerably lower short-term survival rate of our IIM patients compared to the Spanish and Hungarian cohort could be at least partly explained by the age-related difference of studied population. Our cohort comprised of 70% of female IIM patients with a median age at diagnosis of 64 years, while patients from Spain and Hungary (and likewise IIM cohorts from UK, China) were on average 20 years younger (5, 6, 16, 17).

Next, the proportion of patients with cancer among IIM seems important. Sultan et al., who reported in their UK cohort a 95 and 84% 5- and 10-year survival rate, respectively excluded patients with cancer from the analysis (16). When we excluded IIM patients who were diagnosed with cancer a year before, concomitantly or during follow-up from the analysis, the survival outcome was, except in the 1st year after IIM diagnosis, comparable to age and sex matched general population. Our result with a 19% prevalence of cancer and 30% mortality rate during follow-up are comparable to a report by Fang et al. (18) reported a 17% prevalence of IIM associated cancer. Furthermore, during a follow-up 34% of their patients died, with the overall survival rates of 83% (63% in patients with cancer) at 1 year and 74% at 5 years.

Cancer was the most frequent cause of death in our cohort, followed by cardiovascular diseases, and infections. Our results are similar to results from Swedish study in which malignancies, diseases of the circulatory and respiratory system represented the main causes of death (8). Active IIM was the least common cause of death in our cohort, and this finding probably reflects an improved IIM treatment nowadays, with an intensive immunosuppressive treatment enabling us to attain disease remission in a significant proportion of patients, though not so infrequently at the expense of increased risk of infection complications.

Finally, despite the heterogeneity of the study's design and differences in the follow up time makes data comparison of IIM survival based on gross domestic product challenging, it seems there are no major variations in the IIM survival rate between high (4–8) to upper middle (17, 19) or lower income countries (20) over 1 and 5 years.

The major predictors of death in our IIM patients were cancer, cardiac involvement, fever, and age. Clinically evident cardiac involvement has been reported in up to 9% of IIM patients in a large IIM cohort (21), however a recent systematic literature review found a subclinical heart involvement in up to 50% of patients (22). Magnetic resonance imaging was not performed routinely to study cardiac involvement, therefore the 11.5% prevalence of heart involvement in our IIM cohort is probably underestimated, particularly in detecting subclinical changes. The negative prognostic role of heart involvement (disease severity, increased mortality) has been observed already in previous studies (3, 5, 23). Using the improved imaging technique, additional multicentric studies focusing on heart involvement in IIM are warranted.

Interestingly, neither interstitial lung disease nor dysphagia emerged as a significant independent mortality risk factor in our study. The limited number of included patients in our IIM cohort, and the possibility of missing those with the most severe lung involvement (due to early patient death and/or an unfinished diagnostic procedure) may be the reasons for not establishing interstitial lung disease as a poor prognostic marker. However, fever that has not been frequently exposed as a poor prognostic factor, predicted the worse survival of our IIM patients. As fever represent a characteristic of antisynthetase syndrome, one could speculate that the latter might be hiding behind this variable. Antisynthetase syndrome is clinically characterized by frequent interstitial lung disease, and serologically by antisynthetase antibodies (24). Indeed, when evaluating mortality risk factors only in patients without diagnosed cancer, the presence of anti-Jo1 antibody emerged, in addition to cardiac involvement and age, as a factor significantly associated with death. Contrary to some prior reports we did not find any sex related differences nor the significant impact diagnostic delay (i.e., symptom duration time before diagnosis) or intensity of induction treatment (number of immunomodulatory drugs used at induction) on IIM survival (6, 25).

Inclusion of clinically diagnosed IIM represents one of the strengths in our study—we included patients that would be otherwise missed/neglected—e.g., antisynthetase syndrome with isolated interstitial lung involvement. Subclassifying patients in the currently IIM subtypes, addressing therefore also the immune-mediated necrotizing myopathy, represents additional study strength. Though patients were ascertained from the database of two secondary/tertiary centers (and one could assume a bias toward more severe IIM disease cases), we believe, our data are representative for the entire country.

The retrospective study design and a relatively short follow-up period should be seen as study limitation. Even though an exclusion of juvenile IIMs patients could be a study drawback, we believe that survival/mortality of juvenile IIM should be evaluated and interpreted individually, as there are significant differences compared to adult IIMs in baseline characteristics (e.g., patient comorbidities, medications, and smoking status among others). Furthermore, during the follow up, disease activity was assessed clinically, and we did not use the 2016 American College of Rheumatology/European League Against Rheumatism criteria for the response in adult IIM for the assessment of treatment effectiveness and IIM activity (26).

In summary, our study shows substantially worse overall survival of adult patients with IIM compared to matched general population, with cancer representing the leading cause of death in our cohort.

## Data Availability Statement

The raw data supporting the conclusions of this article will be made available by the authors, without undue reservation.

## Ethics Statement

The studies involving human participants were reviewed and approved by Slovenian Ethic Committee. Written informed consent for participation was not required for this study in accordance with the national legislation and the institutional requirements.

## Author Contributions

AH, AV, MK, SG, and IH contributed to the acquisition, analysis, and interpretation of data. MT and ŽR helped to revise the manuscript and gave suggestions. All authors meet the authorship requirements and have read and approved the manuscript for submission.

## Funding

This study was funded by the Slovenian Research Agency (ARRS)—research core funding P3-0314.

## Conflict of Interest

The authors declare that the research was conducted in the absence of any commercial or financial relationships that could be construed as a potential conflict of interest.

## Publisher's Note

All claims expressed in this article are solely those of the authors and do not necessarily represent those of their affiliated organizations, or those of the publisher, the editors and the reviewers. Any product that may be evaluated in this article, or claim that may be made by its manufacturer, is not guaranteed or endorsed by the publisher.

## Footnotes

^1^ https://pxweb.stat.si/SiStat/sl/Podrocja/Index/100/prebivalstvo
